# Predicting Pancreatic Cancer in New‐Onset Diabetes Cohort Using a Novel Model With Integrated Clinical and Genetic Indicators: A Large‐Scale Prospective Cohort Study

**DOI:** 10.1002/cam4.70388

**Published:** 2024-11-11

**Authors:** Yongji Sun, Chaowen Hu, Sien Hu, Hongxia Xu, Jiali Gong, Yixuan Wu, Yiqun Fan, Changming Lv, Tianyu Song, Jianyao Lou, Kai Zhang, Jian Wu, Xiawei Li, Yulian Wu

**Affiliations:** ^1^ Second Affiliated Hospital Zhejiang University School of Medicine Hangzhou Zhejiang China; ^2^ Polytechnic Institute Zhejiang University Hangzhou Zhejiang China; ^3^ Department of General Surgery Hangzhou TCM Hospital Affiliated to Zhejiang Chinese Medical University Zhejiang Hangzhou China; ^4^ Innovation Institute for Artificial Intelligence in Medicine Zhejiang University Hangzhou China; ^5^ Key Laboratory of Cancer Prevention and Intervention, China National Ministry of Education, Cancer Institute Second Affiliated Hospital, Zhejiang University School of Medicine Hangzhou Zhejiang China; ^6^ Cancer Center Zhejiang University Hangzhou Zhejiang China; ^7^ Department of Epidemiology Harvard T.H. Chan School of Public Health Boston Massachusetts USA; ^8^ Department of Surgery, Fourth Affiliated Hospital, International Institutes of Medicine Zhejiang University School of Medicine Zhejiang China; ^9^ Institute of Wenzhou Zhejiang University Zhejiang China; ^10^ School of Public Health and Eye Center The Second Affiliated Hospital, Zhejiang University Hangzhou China; ^11^ School of Public Health Zhejiang University School of Medicine Zhejiang Hangzhou China

**Keywords:** early detection, machine learning, pancreatic cancer, single nucleotide polymorphism

## Abstract

**Introduction:**

Individuals who develop new‐onset diabetes have been identified as a high‐risk cohort for pancreatic cancer (PC), exhibiting an incidence rate nearly 8 times higher than the general population. Hence, the targeted screening of this specific cohort presents a promising opportunity for early pancreatic cancer detection. We aimed to develop and validate a novel model capable of identifying high‐risk individuals among those with new‐onset diabetes.

**Methods:**

Employing the UK Biobank cohort, we focused on those developing new‐onset diabetes during follow‐up. Genetic and clinical characteristics available at registration were considered as candidate predictors. We conducted univariate regression analysis to identify potential indicators and used a 5‐fold cross‐validation method to select optimal predictors for model development. Five machine learning algorithms were used for model development.

**Results:**

Among 12,735 patients with new‐onset diabetes, 100 (0.8%) were diagnosed with PC within 2 years. The final model (area under the curve, 0.897; 95% confidence interval, 0.865–0.929) included 5 clinical predictors and 24 single nucleotide polymorphisms. Two threshold cut‐offs were established: 1.28% and 5.26%. The recommended 1.28% cut‐off, based on model performance, reduces definitive testing to 13% of the total population while capturing 76% of PC cases. The high‐risk threshold is 5.26%. Utilizing this threshold, only 2% of the population needs definitive testing, capturing nearly half of PC cases.

**Conclusions:**

We, for the first time, combined clinical and genetic data to develop and validate a model to determine the risk of pancreatic cancer in patients with new‐onset diabetes using machine learning algorithms. By reducing the number of unnecessary tests while ensuring that a substantial proportion of high‐risk patients are identified, this tool has the potential to improve patient outcomes and optimize healthcare sources.

**Abbreviations**
AUC: area under the curve
BMI: body mass index
Cl: confidence interval
DM: diabetes mellitus
GWAS: genome‐wide association study
ICD: International Classification of Diseases
LR: logistic regression
MLP: multilayer perceptron classifier
NoD: new‐onset diabetes
PC: pancreatic cancer
PCAND: pancreatic cancer‐associated new‐onset diabetes
PRS: polygenic risk scores
RF: Random Forest Classifier
SHAP: SHapley Additive Explanation
SNP: single nucleotide polymorphism
SVC: support vector classification
T2DM: type 2 diabetes mellitus
XGB: extreme gradient boosting classifier

## 1 Background

Pancreatic cancer (PC) is notorious for its dismal prognosis, primarily attributed to the absence of efficient early detection methods [1]. The overwhelming majority of patients with PC are diagnosed at an advanced stage, either with locally advanced disease or distant metastasis, leaving only a small subset of patients eligible for potentially curative surgical intervention [2]. However, only approximately 1.1% of males and 0.7% of females in Europe will develop pancreatic cancer before the age of 75 [3]. This implies that the potential risks of screening the general population might outweigh the potential benefits. Thus, efforts should be focused on developing robust tools for recognizing individuals at a high risk of PC, allowing improved potential for monitoring or secondary screening.

The association between diabetes mellitus (DM) and PC is well recognized. At least half of newly diagnosed patients with PC have DM at the time of diagnosis, and the 3‐year risk of being diagnosed with PC among patients with new‐onset diabetes (NoD) is nearly 8‐fold the expected risk [4]. As pancreatic cancer is responsible for less than 1% of NOD cases [4], universal PC screening in all NoD patients is impractical and might lead to an unfavorable risk/benefit balance [5]. Several studies have endeavored to create predictive models to differentiate pancreatic cancer‐associated new‐onset diabetes (PCAND) from classic type 2 diabetes mellitus (T2DM) [6, 7, 8, 9, 10, 11, 12]. For example, Ash Kieran Clift et al. [11] developed and validated a model to assess pancreatic cancer risk in patients with NoD by considering factors including age, sex, BMI, HbA1c, ALT, creatinine, and other clinical indicators. However, these studies primarily concentrate on clinical factors and neglect the use of genetic information to differentiate between PCAND and T2DM. Furthermore, there is a lack of research that combines both clinical and genetic approaches. Machine learning has gained considerable traction in medicine due to its potential for improving the accuracy of clinical prediction [13, 14, 15]. Cichosz et al. [12], using clinical predictors, built an random forest machine learning model to predict the risk of pancreatic cancer among patients with NoD. Nevertheless, the exploration of which machine learning method is more suitable for distinguishing between PCAND and T2DM in the NoD cohort remains a topic of investigation.

In this study, we sought to identify genetic and clinical predictors that are potentially useful for distinguishing between PCAND and T2DM. Leveraging the selected clinical and genetic indicators, we employed a diverse range of advanced machine learning algorithms to construct predictive models, specifically designed for distinguishing between PCAND and T2DM.

## 2 Materials and Methods

### 2.1 Participants and Study Design

The UK Biobank offers a comprehensive range of health‐related data, encompassing various aspects such as biological measurements [16], lifestyle indicators, biomarkers in blood and urine, body and brain imaging, and genome‐wide genotype information. Detailed descriptions of the study's design, data collection, and processing can be found elsewhere [16, 17].

Patients with T2DM were identified using the International Classification of Diseases, 10th Revision (ICD‐10) codes, obtained from death registries, primary care records, hospital admission data, and self‐reported information. Cancer incidence data (ICD‐10) was collected through linkage with national cancer registries. Cases of type 2 diabetes mellitus were defined based on an ICD‐10 code of E11.X, while cases of pancreatic cancer were defined using an ICD‐10 code of C25. However, our included PC cases also feature the pathological phenotype of neuroendocrine carcinoma.

Participants were split into cases of PCAND and T2DM using the following criteria: PCAND comprised cases where individuals were diagnosed with PC within 24 months after being diagnosed with T2DM, while the T2DM group consisted of individuals who did not develop cancer during the follow‐up period and had a follow‐up duration exceeding 36 months after the initial T2DM diagnosis.

We define the follow‐up as ending upon the occurrence of any of the following events: death or the censorship date based on the hospital data (February 29, 2020).

### 2.2 Clinical Information

We classified clinical variables as indicators of baseline characteristics, physical measures, family history, lifestyle, blood count, and blood biochemistry and collected them during the first assessment visit period (2006–2010). We extracted 2 baseline characteristics indicators (sex, age) and 10 physical measures indicators (pulse rate, diastolic blood pressure, systolic blood pressure, waist circumference, weight, body mass index (BMI), hip circumference, standing height, seated height, sitting height). Besides, we extracted all variables in the categories of blood count and blood biochemistry. For family history, if either the father, mother, or sibling has the disease, the participant is considered to have a family history of that disease. We extracted family history information for three diseases, including family history of diabetes, family history of bowel cancer, and family history of cancer. For lifestyle, we extracted smoking status and alcohol drinker status. Additionally, we included four systemic inflammatory markers that have previously been established as potential correlates of cancer risk [18].

In this study, we included 82 unique clinical variables in total. The list of clinical predictors is shown in Table S1.

### 2.3 Genotyping and SNP Selection

Genome‐wide genetic data is available for 488,000 UK Biobank participants. We conduct research using genotype data. Genotype calling was performed by Affymetrix on two closely related, purpose‐designed arrays. ~50,000 participants were run on the UK BiLEVE Axiom array, and the remaining ~450,000 were run on the UK Biobank Axiom array. The dataset combines results from both arrays, and there are 805,426 markers in the released genotype data. The positions of markers in the data are in GRCh37 coordinates. (more details in https://biobank.ndph.ox.ac.uk/showcase/label.cgi?id=263).

We selected single nucleotide polymorphism (SNP) loci that help distinguish between PCAND and T2DM. We extracted variants of a minor allele frequency ≥ 0.01 and with missing data less than 5%. The selected SNP loci come from two aspects: (1) We extracted SNP loci from 8 pancreatic cancer‐related genes (*SRI* [19], *STAT3* [20], *KRAS* [21], *CDKN2A* [22], *SMAD4* [23], *TP53* [24], *VNN1* [25], *MMP9* [25]) and applied univariate logistic regression to determine whether these loci can be used to distinguish between PCAND and T2DM. We considered SNP loci with a *p* < 0.05 as potentially significant loci. (2) Using logistic regression as the analytical method, we performed genome‐wide association study (GWAS) analysis on 800,000 SNP loci. We considered SNP loci with a *p* < 0.000005 as potentially significant loci.

### 2.4 Dataset Partitions and Imputation

The population was randomly divided into five non‐overlapping subpopulations. We utilized a 5‐fold nested cross‐validation method, where one of the spatially distinct subpopulations was designated as a test set, and the remaining four subpopulations were combined to create a training set. During each of the five cross‐validation iterations, the test set remained untouched while developing the model, and the training set was utilized for prediction model development. For model evaluation, we initially conducted risk value predictions for each individual sample within their corresponding test sets. Subsequently, the predictions generated from the respective test sets were aggregated to perform the final evaluation. Normalization was applied to variables. We presumed that missing data occurred randomly and adopted a KNN Imputer algorithm (*n*_neighbors = 10) to manage the null values in the dataset.

### 2.5 Model Development

The modeling process comprises two primary steps: firstly, the selection of indicators for optimal model performance, and secondly, the utilization of diverse machine learning methods for constructing predictive models.

In the initial step, we approximated the SHapley Additive Explanation (SHAP) values [26] to gain a deeper understanding of the significance of each indicator. Generally, indicators with larger absolute SHAP values hold greater importance in individual predictions. In this study, we calculated the SHAP values using a logistic regression model. Clinical and genetic indicators were assessed separately during the SHAP calculation process. Subsequently, the indicators are sorted in descending order based on their SHAP values. After obtaining the SHAP values for each indicator, the performance of the model is evaluated by utilizing a 5‐fold nested cross‐validation method, considering the inclusion of different numbers of indicators. We selected the combination of indicators that yielded the best performance as the final modeling indicators.

In the second step, we applied five commonly used machine learning algorithms, including logistic regression (LR) [27], support vector classification (SVC) [28], random forest classifier (RF) [29], multilayer perceptron classifier (MLP) [30], and extreme gradient boosting classifier (XGB) [31], to construct our models. Based on the clinical and genetic indicators identified in Step 1, we separately constructed clinical models, genetic models, and combined clinical and genetic models. In all modeling procedures, the time from the registration date to the diabetes diagnosis was incorporated as a covariate.

### 2.6 Model Performance and Clinical Utility

The performance evaluation of the prediction model in the test set involved the assessment of discrimination and calibration. Discrimination was quantified using the area under the receiver operating characteristic curve (AUC) and precision‐recall (PR) curve, while calibration was examined through calibration plots. The model with the most favorable discrimination and calibration performance was selected as the final model. For this final model, we calculated the event rate of pancreatic cancer across predicted risk state percentiles ranging from 0 to 100. Decision curve analysis was used to calculate a clinical net benefit for the final model.

In this study, two distinct threshold cut‐offs were established, namely 1.28% and 5.26%. The threshold of 1.28% was defined as the optimal risk threshold, derived from a comprehensive assessment that aimed to maximize both sensitivity and specificity concurrently. On the other hand, the threshold of 5.26% was designated as the high‐risk threshold. In Figure 4a, we observed a notably high pancreatic cancer event rate among the top 2% of the population. Consequently, restricting PC screening to only this top 2% segment sets the threshold for screening at 5.26%. Additionally, a set of four universally applied threshold values (0.5%, 1%, 5%, and 10%) were also established to further augment the efficacy and applicability of our findings. We then calculated clinically relevant metrics such as sensitivity, specificity, positive predictive value, negative predict value, and accuracy over multiple risk thresholds.

## 3 Results

### 3.1 Study Population and Study Overview

**FIGURE 1:**
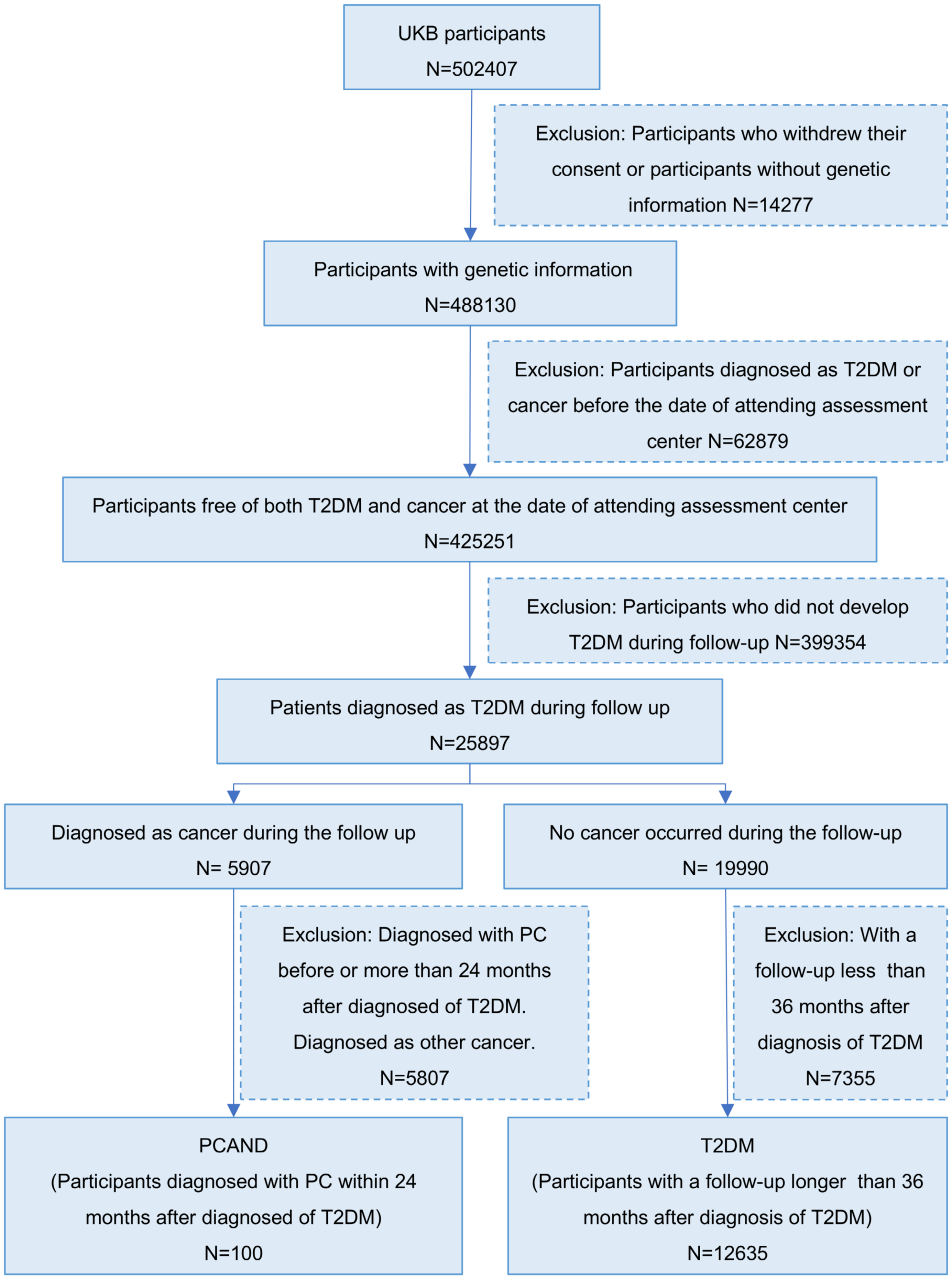
Participants flow diagram.

Figure 1 shows our data assembly process. 502,407 participants were enrolled into the UK Biobank cohort between 2006 and 2010. Participants who withdrew their consent (*n* = 40) or participants without genetic information (*n* = 14,237) were also excluded from the study, leaving 488,130 participants. We specifically considered cases in which individuals were free of both type 2 diabetes mellitus (T2DM) and cancer at the time of their attendance at the assessment center, leaving 425,251 participants. Subsequently, we included individuals who developed T2DM during the follow‐up period, leaving 25,897. Participants were then categorized into two groups, PCAND and T2DM. There are 100 participants diagnosed with PC within 24 months after diagnosis of T2DM cases, and 12,635 participants did not develop cancer during the follow‐up period and had a follow‐up duration exceeding 36 months after the initial T2DM diagnosis. The median interval between the diagnosis of new‐onset diabetes and the subsequent diagnosis of pancreatic cancer among these 100 PCAND cases was 33 (interquartile range 9.25–175.75) days. Additionally, the median interval between attending the assessment center and the diagnosis of new‐onset diabetes among these PCAND and T2DM cases was 4.49 (interquartile range 2.45–6.36) years.

### 3.2 Clinical Predictors Selection

**FIGURE 2:**
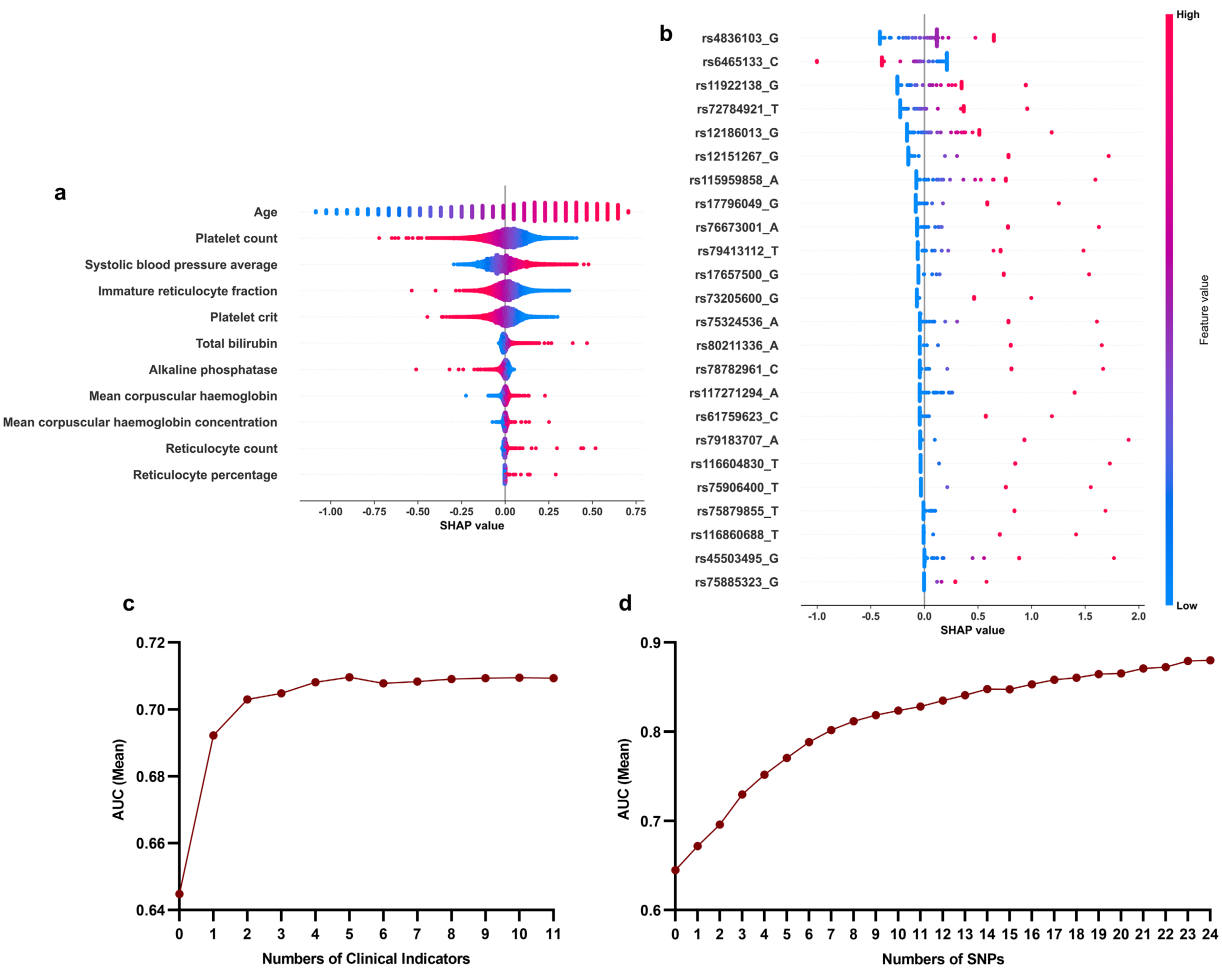
Exploring the significance of diverse indicators and evaluating model performance across variable indicator sets. (a, b) Beeswarm plot of features in the model. Elements are ranked from top to bottom by the absolute value of the mean SHAP value. (a) illustrates the SHAP values of 11 clinical predictors, while (b) depicts the SHAP values of 24 SNPs. (c, d), Assessment of model performance with varying numbers of indicators. The vertical axis portrays the mean value of the area under the curve (AUC), derived from a 5‐fold cross‐validation of the model. The model with the 0 indicator represents a model that is built solely using the duration of follow‐up from the registration date to the date of diabetes diagnosis as a covariate. (c) illustrates the performance of clinical models, while (d) depicts the performance of genetic models.

In this study, we assessed a comprehensive set of 82 candidate clinical predictors for their potential in predicting diabetes onset (Table S2). Through univariate logistic regression analysis, we identified 11 variables with *p* < 0.05, including 1 anthropometry, 1 physical measure, 5 blood biochemistry, and 4 blood count variables. To gain further insights into the significance of each clinical indicator, we employed the calculation of SHAP values, as depicted in the accompanying Figure 2a. Notably, our analysis revealed that age exerted the greatest impact among the variables, while reticulocyte percentage exhibited the least impact. During the modeling process, we discovered that the model achieved optimal performance when integrating the top five clinical indicators, as demonstrated in Figure 2c. Finally, we proceeded to construct our clinical model by incorporating age at recruitment, platelet count, systolic blood pressure, immature reticulocyte fraction, and platelet crit.

### 3.3 SNP Locus Selection

We identified a total of 611,257 single nucleotide polymorphisms meeting the criteria of having a minor allele frequency (MAF) of at least 0.01 and missing data below 5%. Within the scope of eight genes associated with pancreatic cancer, we detected two variants, namely rs6465133 (*SRI*) (*p* = 0.001) and rs61759623 (*KRAS*) (*p* = 0.005), which exhibit the potential to differentiate between PCAND and T2DM. Employing genome‐wide association study analysis, we further identified an additional set of 22 SNPs contributing to the discrimination between PCAND and T2DM. Table S3 presents the complete list of these SNPs.

To gain deeper insights into the significance of each indicator, we employed SHAP values, as illustrated in Figure 2b. Throughout the modeling process, we discovered that optimal performance was achieved by integrating all 24 SNPs (Figure 2d).

### 3.4 Model Performance

**FIGURE 3:**
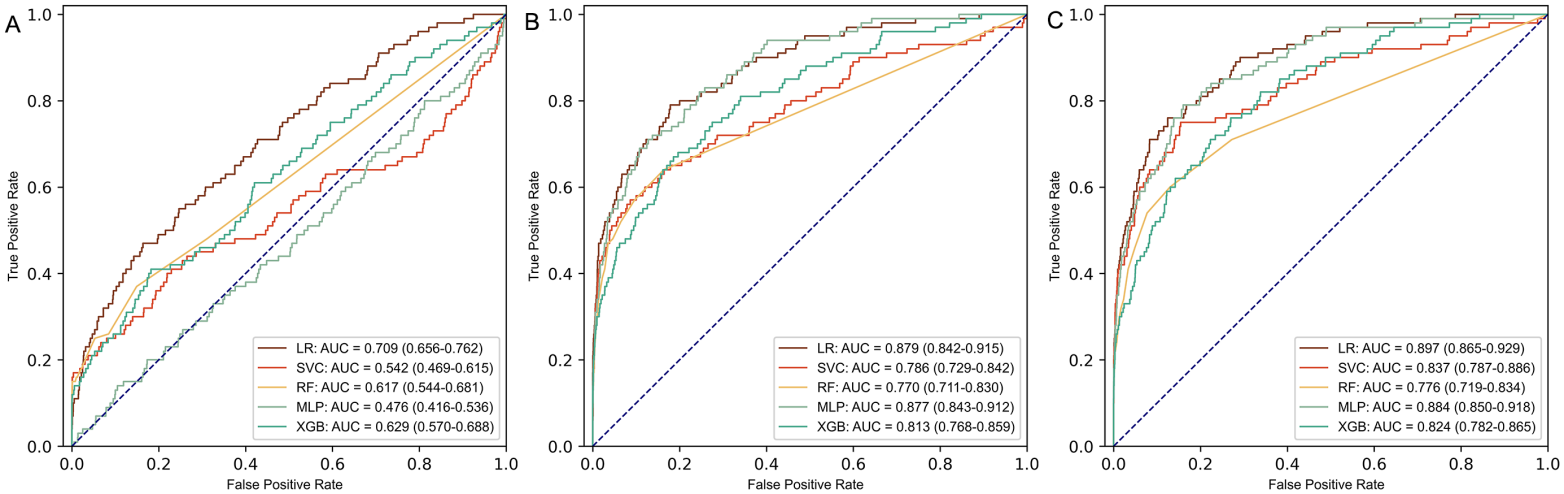
Receiver operator curve of different models. Subfigure (A) illustrates the performance of five clinical models, subfigure (B) depicts the performance of five genetic models, and subfigure (C) represents the performance of five clinical + genetic models.

Notably, the genetic model exhibited relatively strong discrimination performance across different algorithms (AUC > 0.75 for all), particularly in the LR model (0.879, 95% confidence interval [Cl] 0.842–0.915) and MLP model (0.877, 95% CI 0.843–0.912) (Figure 3B). In contrast, the clinical model demonstrated lower performance compared to the genetic model. Logistic regression yielded the best performance for the clinical model (0.709, 95% CI 0.656–0.762), while the other modeling algorithms fell short of satisfactory results (AUC < 0.65 for all) (Figure 3A). Additionally, the integration of genetic and clinical information yielded superior performance compared to employing a single omics. Notably, the LR algorithm demonstrated the highest performance (0.897, 95% CI 0.865–0.929) (Figure 3C).

Regarding the PR curve, the genetic model demonstrates significantly superior performance compared to the clinical model. However, the combined genetic and clinical model does not exhibit a significant improvement over the genetic model alone (Figure S1). Regarding calibration, the relevant results are presented in Figure S1. In the domain of clinical modeling, our investigation indicates that the LR model exhibits commendable calibration, whereas the performance of the remaining four algorithms was found to be unsatisfactory (Figure S2a). In the realm of genetic modeling, all five algorithms demonstrated favorable performance, with notable performance observed in the LR and MLP models (Figure S2b). Moreover, when integrating genetic and clinical information, we observed that, excluding XGB, the other four algorithms achieved well‐calibrated results in the test set (Figure S2c).

**FIGURE 4:**
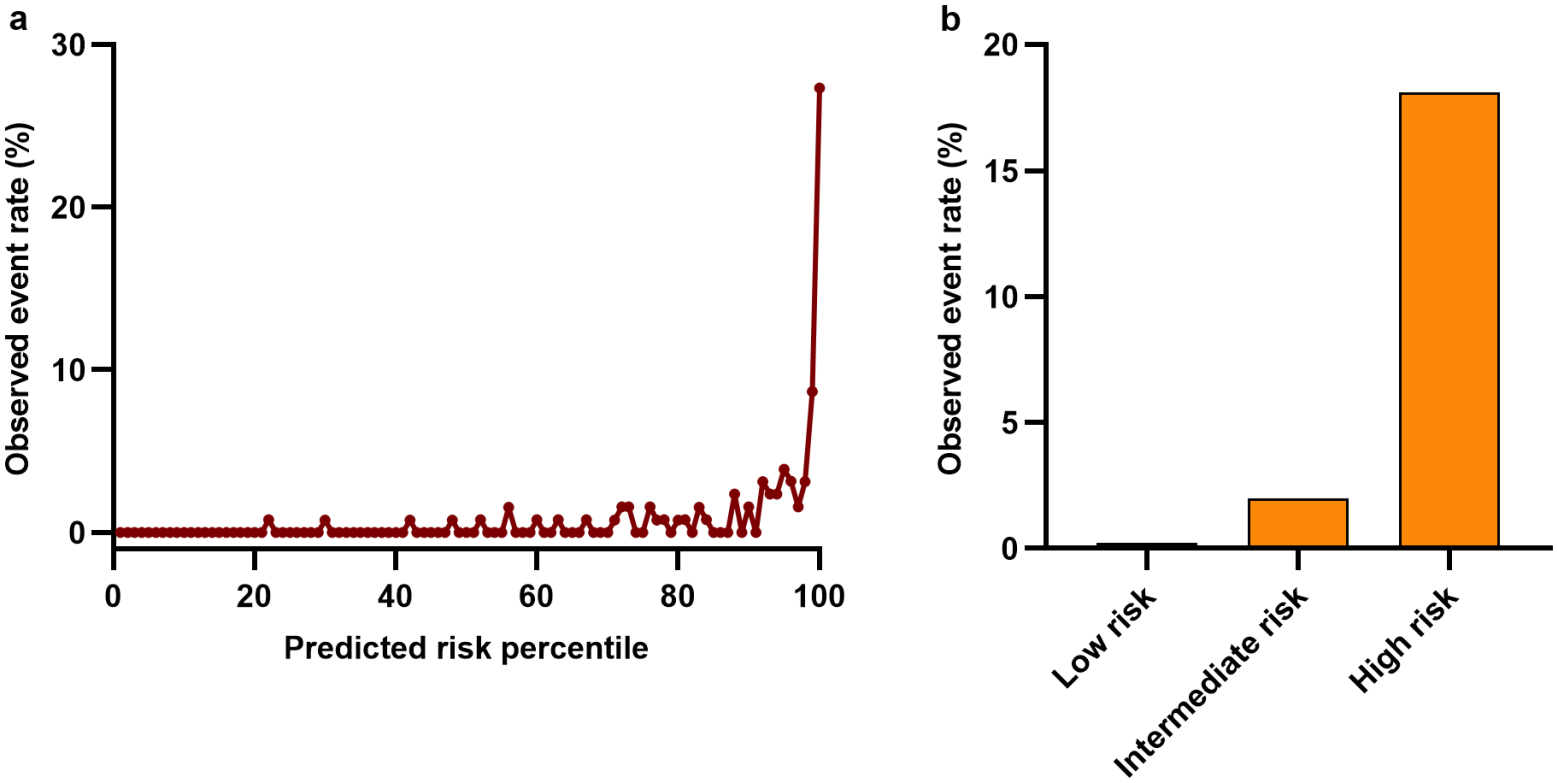
The relationship between the observed event frequency of pancreatic cancer and the predicted risk status. (a) illustrates the observed event frequency of pancreatic cancer plotted against predicted risk state percentiles over the entire study population; (b) depicts the pancreatic cancer event rate of three risk groups.

According to the extensive evaluation of discrimination and calibration performance, our ultimate model integrates genetic and clinical data and employs logistic regression for its development. In order to delve deeper into the potential of predictive risk status as an indicator of pancreatic cancer susceptibility, we conducted an investigation into its association with incident event rates (Figure 4a). Our investigation revealed a positive correlation between higher predicted risk statuses and an elevated incidence of pancreatic cancer. Additionally, notable escalations in pancreatic cancer incidence were observed within the 99th (8.66%) and 100th (27.34%) percentiles.

### 3.5 Performance Translates to Clinical Utility

While model performance is critical, the clinical utility of any risk model depends on the choice of adequate thresholds for interventions. Adequate clinical decision thresholds directly depend on the benefits and harms of interventions and disease prevalence. Therefore, we calculated the decision curve (Figure S3) to estimate the clinical benefit of our final model. We found that within a large range of thresholds (0–0.76), clinical decision‐making would benefit from using the decision model.

Table 1 presents the sensitivity, specificity, positive predictive value, negative predictive value, and accuracy for six different probability cut‐offs (1.28%, 5.26%, 0.5%, 1%, 5%, and 10%). For example, if the risk threshold for further PC screening was set at 1.28%, 13% of the new‐onset diabetes population would undergo screening, and the sensitivity, specificity, positive predictive value, negative predictive value, and accuracy of the model would be 76.0%, 87.5%, 4.6%, 99.8%, and 87.4%, respectively. For a risk threshold of 5.26%, 2.0% of the new‐onset diabetes population would undergo screening, and the corresponding sensitivity, specificity, positive predictive value, negative predictive value, and accuracy would be 46.0%, 98.4%, 18.1%, 99.6%, and 97.9%. Using the thresholds of 1.28% and 5.26%, we stratified the population into low, intermediate, and high‐risk categories. Among individuals classified as low risk, a remarkably low incidence rate of pancreatic cancer events was observed (0.2%) (Figure 4b; Table S4). In the intermediate risk group, the incidence rate was 2.0%. In the high‐risk group, a notably high incidence rate of pancreatic cancer events was observed (18.1%).

**TABLE 1:** Model diagnostic performance at different predicted probability cut‐offs.

Probability cut‐off	Distinct cut‐offs		Common cut‐offs			
	1.28%	5.26%	0.50%	1%	5%	10%
Sensitivity	0.760	0.460	0.900	0.770	0.460	0.300
Specificity	0.875	0.984	0.687	0.837	0.982	0.995
PPV^a^	0.046	0.181	0.022	0.036	0.165	0.326
NPV^b^	0.998	0.996	0.999	0.998	0.996	0.994
Accuracy	0.874	0.979	0.688	0.837	0.977	0.99
Need screening	0.130	0.020	0.318	0.167	0.022	0.007

^a^ Positive predictive value.

^b^ Negative predictive value.

Since the inclusion of clinical variables results in minimal improvement compared to genetic variables alone, we also conducted this analysis using the gene‐only model, as illustrated in Figures S3 and S4 and Tables S5 and S6.

## 4 Discussion

Patients with new‐onset diabetes (NoD) have been identified as a high‐risk group for pancreatic cancer (PC) [4]. However, conducting universal PC screening in all NoD patients is impractical due to the fact that the majority of these patients have classic type 2 diabetes mellitus (T2DM) rather than pancreatic cancer‐associated new‐onset diabetes (PCAND) [32]. As PC only accounts for approximately 0.8% of NoD cases [4], effectively identifying potential PCAND cases within the large base of NoD patients and distinguishing it them from T2DM remains a major challenge.

To address this challenge, previous studies have attempted to develop predictive models relying on clinical data to differentiate between PCAND and T2DM [6, 7, 8, 9, 10, 11, 12]. Among these models, it has been found that the one with the highest discriminative ability achieves an area under the curve (AUC) of 0.87 [6]. Regrettably, these models have shown suboptimal performance when applied to external validation datasets; the same model [6] only obtained an AUC of 0.75 [33]. The challenges encountered in previous models could potentially be attributed to the exclusive focus on clinical factors while disregarding microscopic biological factors. A recent investigation revealed that the integration of polygenic risk scores (PRS) derived from prior studies into a risk model consisting of conventional clinical features resulted in an enhanced PC discriminative capacity with an AUC of 0.83 [34]. The lower AUC value could potentially be attributed to the fact that this model was trained using data from the entire population rather than exclusively focusing on individuals with NoD. In this study, we employed advanced machine learning algorithms and, innovatively, integrated clinical and genetic data to construct predictive models for distinguishing PCAND from T2DM. Remarkably, our final model demonstrated exceptional performance with an AUC of 0.897 (95% CI 0.865–0.929), surpassing the performance of existing models [6, 7, 8, 9, 10, 11, 12]. Our finding indicated significant potential in leveraging machine learning to integrate genetic and clinical factors and apply them to scenarios aimed at distinguishing between PCADN and T2DM.

Genetic factors play a pivotal role in the etiology and progression of PCAND. Our team's previously published articles explored the genetic characteristics of PCAND and T2DM, uncovering potential molecular markers such as *SRI* [19], *VNN1* [25], and *MMP9* [25] for distinguishing between the two conditions. Indeed, our past research primarily focused on the central dogma and characteristics of genes without delving into the deeper level of gene sequences. Nowadays, genome‐wide association studies (GWAS) have emerged as a potent and hypothesis‐free approach for identifying common alleles that influence the risk of diseases [34, 35]. In recent years, several single nucleotide polymorphisms (SNPs) with compelling associations to PC risk have been reported [36, 37]. However, to date, no investigation has explored the relationship between susceptibility variants of T2DM and PCAND in individuals with NoD. Several factors contribute to this research gap, and one notable aspect is that studies on newly diagnosed diabetes have predominantly relied on retrospective cohorts, such as The Health Improvement Network [8], which does not include genetic sequencing as a routine examination procedure. In this study, we identified a set of 24 SNPs, comprising both well‐acknowledged pancreatic cancer‐related genes and SNPs identified through GWAS analysis, which exhibit the potential to differentiate between T2DM and PCAND. To our knowledge, this study represents the first attempt to identify SNP markers that can differentiate between PCAND and T2DM in the NoD cohort.

In clinical applications, this study found that PCAND accounted for approximately 0.79% of the total sample, consistent with previous research [4, 38]. This indicates that the ratio between PC and T2DM is representative of the general population. Besides, the identification of an optimal probability cut‐off for definitive screening holds significant importance. Generally, cutoffs with higher sensitivity tend to exhibit a lower positive predictive value. For instance, high‐sensitivity cutoffs can effectively rule out pancreatic cancer. However, the low positive predictive value may render confirmatory testing—such as endoscopic ultrasound or magnetic resonance imaging—costly for population‐based screening programs. Furthermore, the low prevalence of pancreatic cancer within the cohort exacerbates the decline in positive predictive value as sensitivity increases. A recent study indicated that, for screening new‐onset diabetes as a means of detecting pancreatic cancer to be cost‐effective, a baseline risk of 2.5% in a high‐risk cohort is necessary [32].

In this study, we have established two distinct threshold cut‐offs, namely 1.28% and 5.26%. The threshold of 1.28% serves as our recommended cut‐off and is determined based on the model's sensitivity and specificity performance. By using this threshold, we can reduce the number of individuals requiring definitive testing to only 13.0% of the total NoD population while still capturing 76% of all PC‐associated diabetes cases. Notably, among the predicted positive samples, pancreatic cancer accounts for 4.6%, whereas in the entire NoD population, the incidence of pancreatic cancer remains as low as 0.8%. Conversely, the threshold of 5.26% is defined as the high‐risk threshold, as there is a significant rise in pancreatic cancer incidence beyond it. By utilizing a 5.26% predicted risk of PC as the threshold for proceeding with definitive testing, only 2.0% of the entire NoD population would need to undergo definitive testing, yet nearly half of all cases of PC‐associated diabetes within this population would be captured. By leveraging our predictive model, we stratified the population into low, intermediate, and high‐risk categories. Consequently, individuals classified as low‐risk for PC do not require further screening. Regular follow‐up screening is advised for those categorized as intermediate risk. These individuals are advised to continue annual surveillance unless clinically indicated for a shorter follow‐up interval due to detected abnormalities. Intensive follow‐up screening is strongly recommended for high‐risk individuals, involving a shorter surveillance interval of 3–6 months. For patients with new‐onset diabetes classified as intermediate or high risk for PC, a follow‐up monitoring period of 3 years is recommended to determine whether the diabetes is related to pancreatic cancer.

The present investigation is subject to several potential limitations. First, the training and validation procedures of the model solely relied on the UKB dataset, lacking dependable external validation measures to establish conclusive assessments. Second, this study may have issues with overfitting due to the large number of variables and relatively small sample size. Third, it is worth noting that the UKB database predominantly comprises individuals of white ethnicity, thereby necessitating further investigations to ascertain the generalizability of the study findings to other ethnic groups. Fourth, it is important to acknowledge that genetic sequencing does not currently constitute a routine clinical diagnostic and management practice, thereby presenting an obstacle to the practical application of this model. Fifth, this study employed clinical indicators prior to the diagnosis of diabetes. While the clinical status before the diabetes diagnosis effectively differentiates between PCAND and T2DM, the model's performance in this study was inferior to that of most previous models [6, 7, 8, 10, 11, 12]. Sixth, along with many previous studies [6, 7, 8, 9, 10, 11, 12], our model lacks data on the stage of pancreatic cancer at the time of diagnosis, a critical determinant of survival.

## 5 Conclusions

In summary, we have successfully developed and validated a prediction tool with the objective of identifying individuals at a heightened risk of PC within the vast cohort of individuals diagnosed with NoD. This model enables the stratification of NoD populations into low, medium, and high‐risk groups, allowing for tailored follow‐up strategies for each category. This approach not only has the potential to improve patient outcomes but also to reduce healthcare costs significantly.

## Author Contributions

**Yongji Sun:** conceptualization (lead), methodology (lead), writing – original draft (lead). **Chaowen Hu:** software (lead), writing – original draft (supporting). **Sien Hu:** data curation (lead), methodology (supporting), writing – original draft (supporting). **Hongxia Xu:** writing – review and editing (equal). **Jiali Gong:** writing – review and editing (equal). **Yixuan Wu:** software (supporting). **Yiqun Fan:** investigation (equal). **Changming Lv:** investigation (equal). **Tianyu Song:** data curation (supporting). **Jianyao Lou:** investigation (supporting). **Kai Zhang:** investigation (supporting). **Jian Wu:** funding acquisition (equal), project administration (equal), resources (equal), supervision (equal), writing – review and editing (equal). **Xiawei Li:** project administration (equal), writing – review and editing (equal). **Yulian Wu:** funding acquisition (equal), project administration (equal), resources (equal), supervision (equal), writing – review and editing (equal).

## Ethics Statement

The data collection in UK Biobank was approved by the NHS National Research Ethics Service (ref 11/NW/0382). All data sets used in this data obtained fully informed consent from participants.

## Conflicts of Interest

The authors declare no conflicts of interest.

## Supporting information


Data S1.

## Data Availability

This research was conducted using the UK Biobank Resource under application number [91799]. The source code for the analysis is available at https://github.com/jhrsya/pancreatic_cancer_multiomics.
