# Supplementary material for: Predicting Pancreatic Cancer in New‐Onset Diabetes Cohort Using a Novel Model With Integrated Clinical and Genetic Indicators: A Large‐Scale Prospective Cohort Study
Source: Cancer Med. 2024 Nov 11;13(21):e70388. doi: 10.1002/cam4.70388 (PMC11551786; doi:10.1002/cam4.70388)
Supplement: Supplementary file 1 — Data S1. [file CAM4-13-e70388-s001.docx]

**Supplementary figures and tables**

**Figure S1. Precision-Recall curve of different models**

**Figure S2. Calibration plots of observed versus predicted risk**

**Figure S3. Decision curve analysis of different models**

**Figure S4. The relationship between the observed event frequency of pancreatic cancer and the predicted risk status in the genetic model**

**Table S1. Details on clinical predictors in this study**

**Table S2. Participants' characteristics and effect of pancreatic cancer risk in the univariable logistic regression analysis**

**Table S3. Summary results of SNPs used**

**Table S4. The distribution of three risk groups among the entire population**

**Table S5. Genetic model diagnostic performance at different predicted probability cut-offs**

**Table S6. The distribution of three risk groups among the entire population based on genetic model**


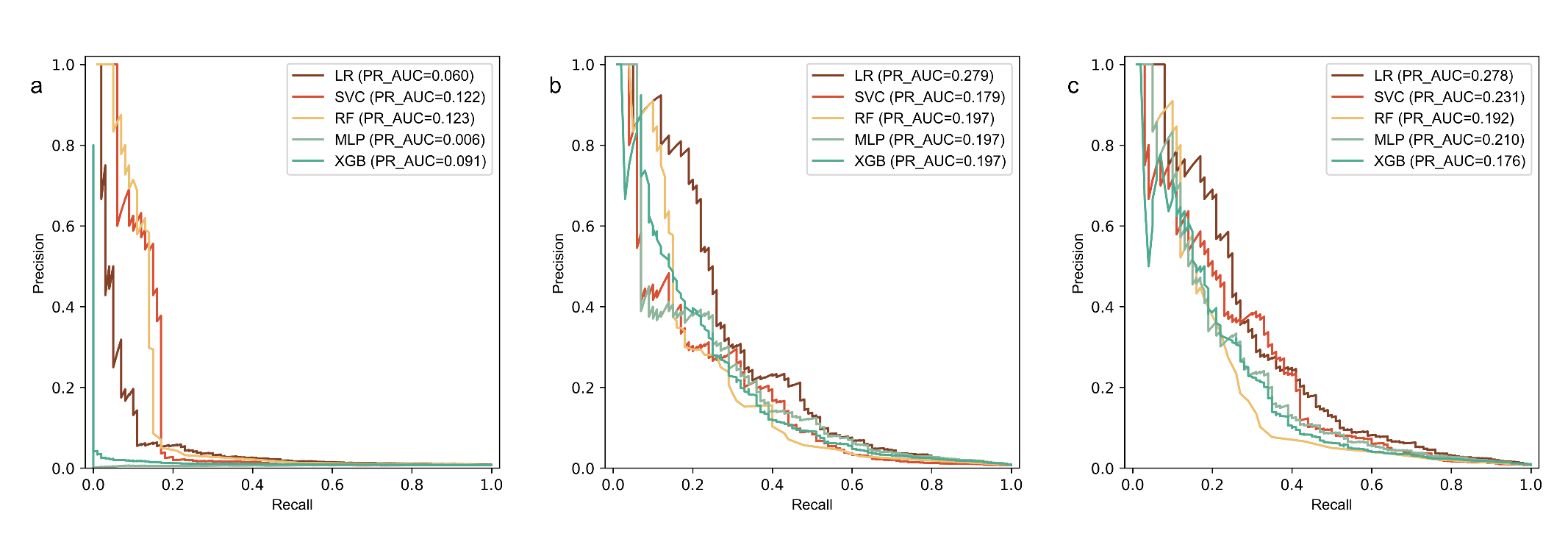


**Figure S1. Precision-Recall curve of different models.** Subfigure (a) illustrates the performance of five clinical models, subfigure (b) depicts the performance of five genetic models, and subfigure (c) represents the performance of five clinical + genetic models.


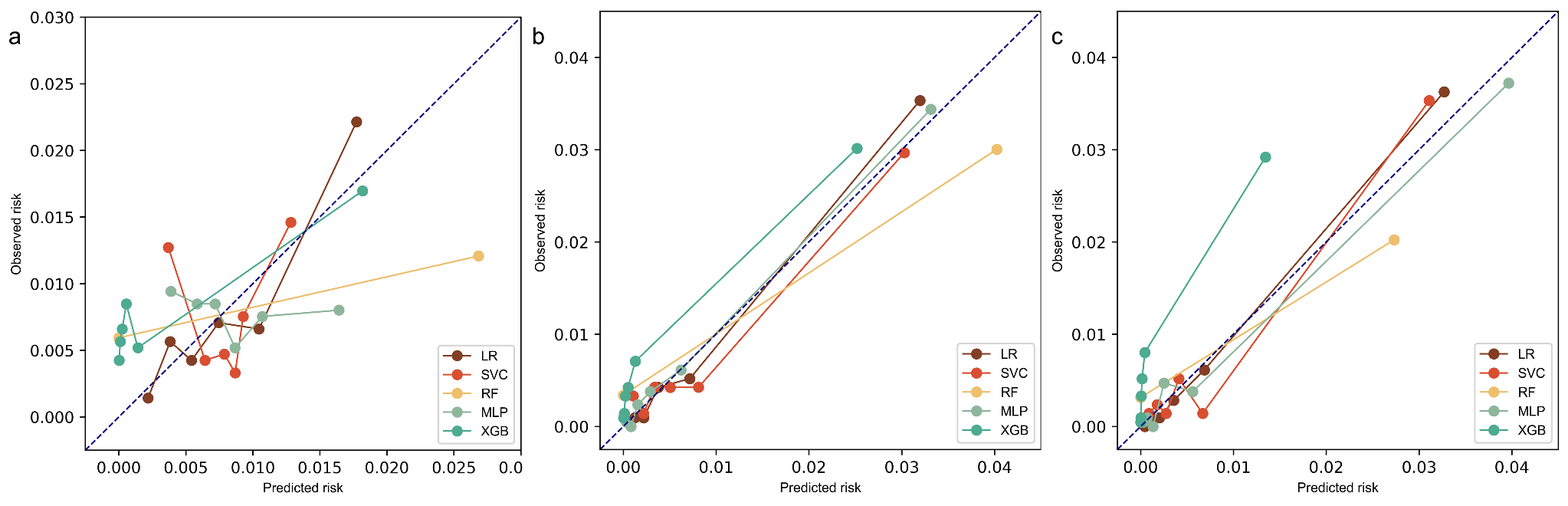


**Figure S2. Calibration plots of observed versus predicted risk.** Subfigure (a) illustrates the performance of five clinical models, subfigure (b) depicts the performance of five genetic models, and subfigure (c) represents the performance of five clinical + genetic models


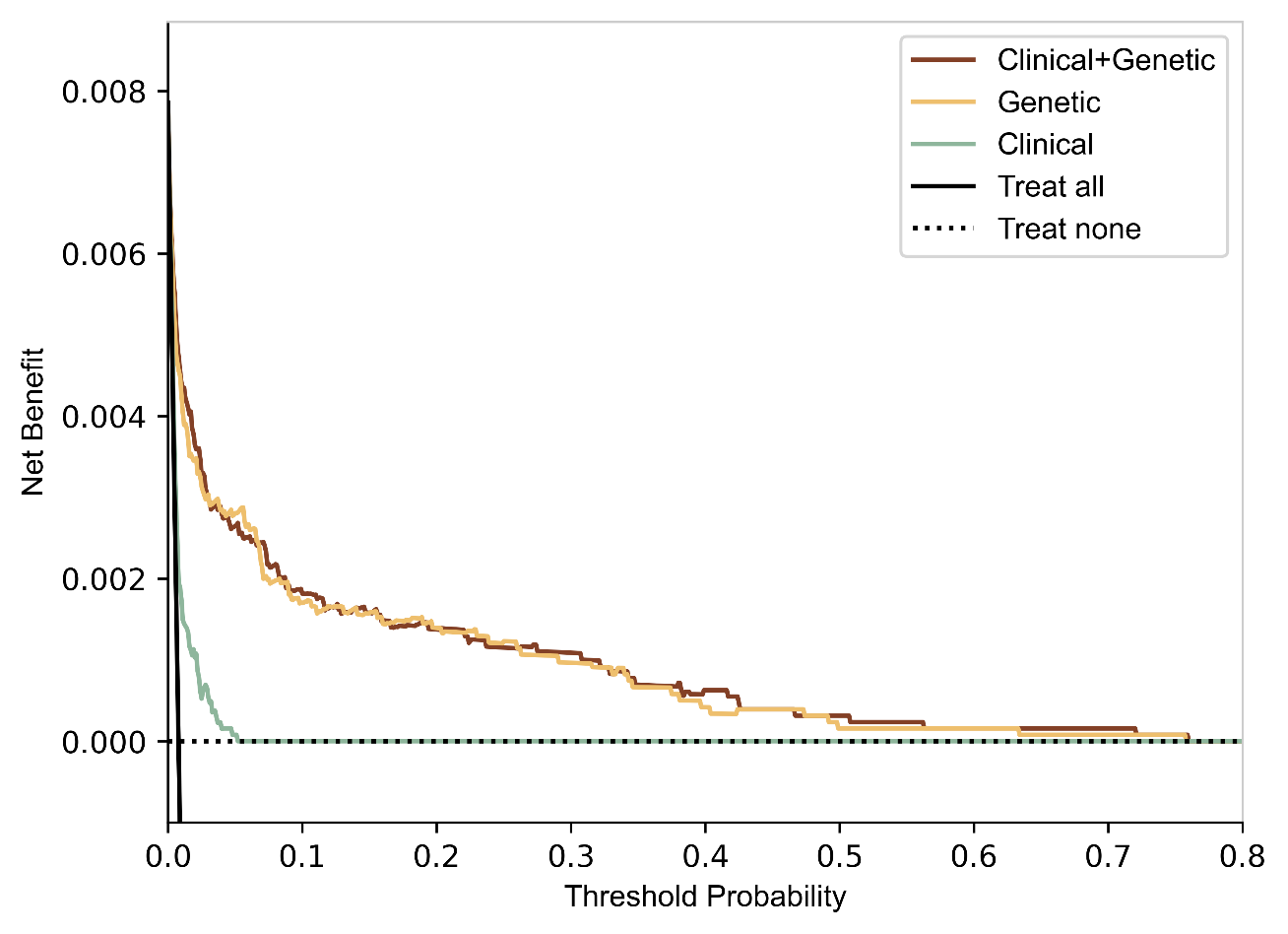


**Figure S3. Decision curve analysis of different models.**


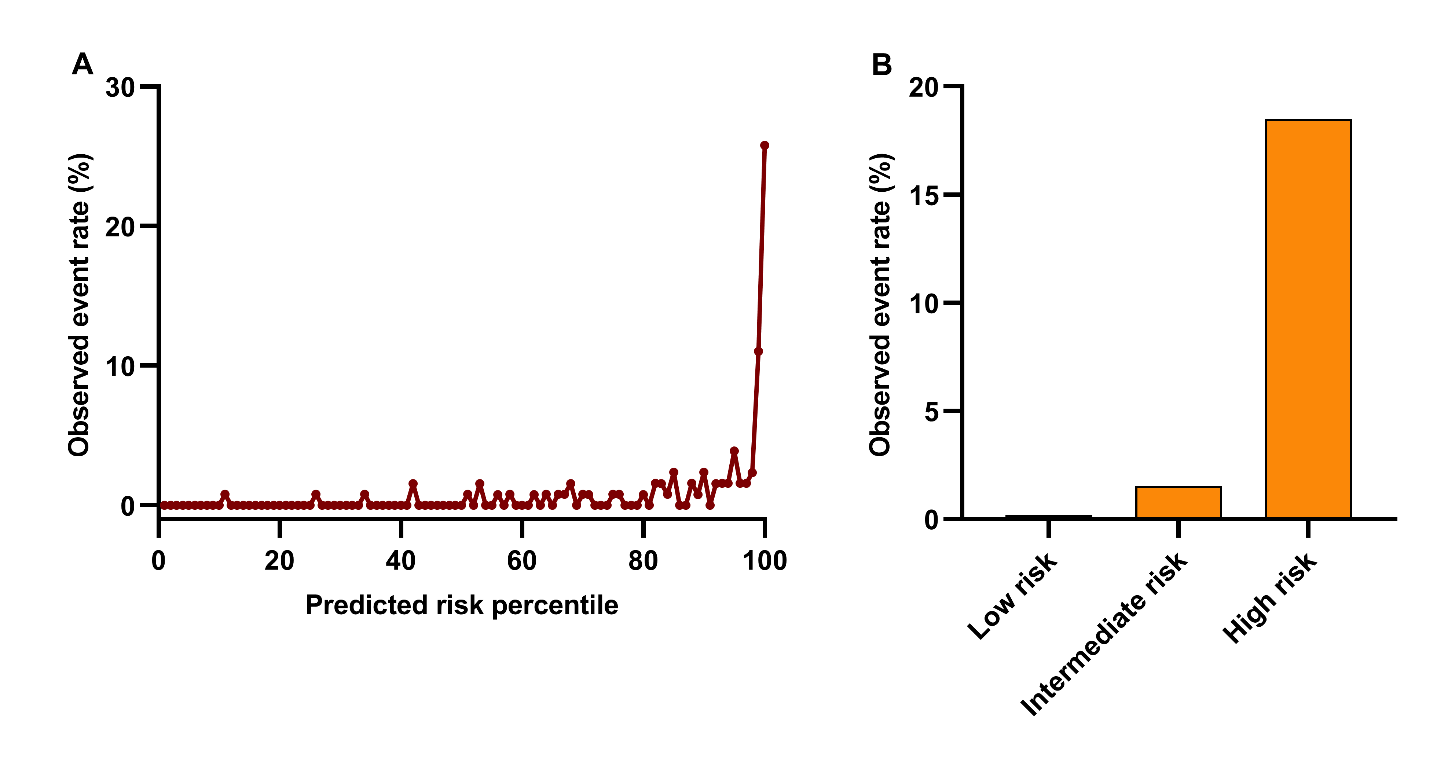


**Figure S4. The relationship between the observed event frequency of pancreatic cancer and the predicted risk status in the genetic model.** Subfigure (a) illustrates the Observed event frequency of pancreatic cancer plotted against predicted risk state percentiles over the entire study population, subfigure (b) depicts pancreatic cancer event rate of three risk groups.

| **Table S1. Details on clinical predictors in this study.** | | | |
| --- | --- | --- | --- |
| Predictor | Class | Source | Field ID |
| Age at recruitment | Continuous | baseline characteristics | 21022 |
| Sex | Binary | baseline characteristics | 31 |
| Pulse rate, automated reading | Continuous | Physical assessment at recruitment | 102 |
| Diastolic blood pressure, automated reading | Continuous | Physical assessment at recruitment | 4079 |
| Systolic blood pressure, automated reading | Continuous | Physical assessment at recruitment | 4080 |
| Waist circumference | Continuous | Physical assessment at recruitment | 48 |
| Weight | Continuous | Physical assessment at recruitment | 21002 |
| Body mass index (BMI) | Continuous | Physical assessment at recruitment | 21001 |
| Hip circumference | Continuous | Physical assessment at recruitment | 49 |
| Standing height | Continuous | Physical assessment at recruitment | 50 |
| Seated height | Continuous | Physical assessment at recruitment | 51 |
| Sitting height | Continuous | Physical assessment at recruitment | 20015 |
| Systemic immune-inflammation index (SII) | Continuous | Blood count assessment at recruitment | 30140, 30080,30120 |
| neutrophil-to-lymphocyte ratio (NLR) | Continuous | Blood count assessment at recruitment | 30140, 30120 |
| platelet-to-lymphocyte ratio (PLR) | Continuous | Blood count assessment at recruitment | 30080, 30120 |
| lymphocyte-to-monocyte ratio (LMR) | Continuous | Blood count assessment at recruitment | 30120, 30130 |
| Basophill count | Continuous | Blood count assessment at recruitment | 30160 |
| Basophill percentage | Continuous | Blood count assessment at recruitment | 30220 |
| Eosinophill count | Continuous | Blood count assessment at recruitment | 30150 |
| Eosinophill percentage | Continuous | Blood count assessment at recruitment | 30210 |
| Haematocrit percentage | Continuous | Blood count assessment at recruitment | 30030 |
| Haemoglobin concentration | Continuous | Blood count assessment at recruitment | 30020 |
| High light scatter reticulocyte count | Continuous | Blood count assessment at recruitment | 30300 |
| High light scatter reticulocyte percentage | Continuous | Blood count assessment at recruitment | 30290 |
| Immature reticulocyte fraction | Continuous | Blood count assessment at recruitment | 30280 |
| Lymphocyte count | Continuous | Blood count assessment at recruitment | 30120 |
| Lymphocyte percentage | Continuous | Blood count assessment at recruitment | 30180 |
| Mean corpuscular haemoglobin | Continuous | Blood count assessment at recruitment | 30050 |
| Mean corpuscular haemoglobin concentration | Continuous | Blood count assessment at recruitment | 30060 |
| Mean corpuscular volume | Continuous | Blood count assessment at recruitment | 30040 |
| Mean platelet (thrombocyte) volume | Continuous | Blood count assessment at recruitment | 30100 |
| Mean reticulocyte volume | Continuous | Blood count assessment at recruitment | 30260 |
| Mean sphered cell volume | Continuous | Blood count assessment at recruitment | 30270 |
| Monocyte count | Continuous | Blood count assessment at recruitment | 30130 |
| Monocyte percentage | Continuous | Blood count assessment at recruitment | 30190 |
| Neutrophill count | Continuous | Blood count assessment at recruitment | 30140 |
| Neutrophill percentage | Continuous | Blood count assessment at recruitment | 30200 |
| Nucleated red blood cell count | Continuous | Blood count assessment at recruitment | 30170 |
| Nucleated red blood cell percentage | Continuous | Blood count assessment at recruitment | 30230 |
| Platelet count | Continuous | Blood count assessment at recruitment | 30080 |
| Platelet crit | Continuous | Blood count assessment at recruitment | 30090 |
| Platelet distribution width | Continuous | Blood count assessment at recruitment | 30110 |
| Red blood cell (erythrocyte) count | Continuous | Blood count assessment at recruitment | 30010 |
| Red blood cell (erythrocyte) distribution width | Continuous | Blood count assessment at recruitment | 30070 |
| Reticulocyte count | Continuous | Blood count assessment at recruitment | 30250 |
| Reticulocyte percentage | Continuous | Blood count assessment at recruitment | 30240 |
| White blood cell (leukocyte) count | Continuous | Blood count assessment at recruitment | 30000 |
| Alanine aminotransferase | Continuous | Blood biochemistry assessment at recruitment | 30620 |
| Albumin | Continuous | Blood biochemistry assessment at recruitment | 30600 |
| Alkaline phosphatase | Continuous | Blood biochemistry assessment at recruitment | 30610 |
| Apolipoprotein A | Continuous | Blood biochemistry assessment at recruitment | 30630 |
| Apolipoprotein B | Continuous | Blood biochemistry assessment at recruitment | 30640 |
| Aspartate aminotransferase | Continuous | Blood biochemistry assessment at recruitment | 30650 |
| C-reactive protein | Continuous | Blood biochemistry assessment at recruitment | 30710 |
| Calcium | Continuous | Blood biochemistry assessment at recruitment | 30680 |
| Cholesterol | Continuous | Blood biochemistry assessment at recruitment | 30690 |
| Creatinine | Continuous | Blood biochemistry assessment at recruitment | 30700 |
| Cystatin C | Continuous | Blood biochemistry assessment at recruitment | 30720 |
| Direct bilirubin | Continuous | Blood biochemistry assessment at recruitment | 30660 |
| Gamma glutamyltransferase | Continuous | Blood biochemistry assessment at recruitment | 30730 |
| Glucose | Continuous | Blood biochemistry assessment at recruitment | 30740 |
| Glycated haemoglobin (HbA1c) | Continuous | Blood biochemistry assessment at recruitment | 30750 |
| HDL cholesterol | Continuous | Blood biochemistry assessment at recruitment | 30760 |
| IGF-1 | Continuous | Blood biochemistry assessment at recruitment | 30770 |
| LDL direct | Continuous | Blood biochemistry assessment at recruitment | 30780 |
| Lipoprotein A | Continuous | Blood biochemistry assessment at recruitment | 30790 |
| Oestradiol | Continuous | Blood biochemistry assessment at recruitment | 30800 |
| Phosphate | Continuous | Blood biochemistry assessment at recruitment | 30810 |
| Rheumatoid factor | Continuous | Blood biochemistry assessment at recruitment | 30820 |
| SHBG | Continuous | Blood biochemistry assessment at recruitment | 30830 |
| Testosterone | Continuous | Blood biochemistry assessment at recruitment | 30850 |
| Total bilirubin | Continuous | Blood biochemistry assessment at recruitment | 30840 |
| Total protein | Continuous | Blood biochemistry assessment at recruitment | 30860 |
| Triglycerides | Continuous | Blood biochemistry assessment at recruitment | 30870 |
| Urate | Continuous | Blood biochemistry assessment at recruitment | 30880 |
| Urea | Continuous | Blood biochemistry assessment at recruitment | 30670 |
| Vitamin D | Continuous | Blood biochemistry assessment at recruitment | 30890 |
| Family history of bowel cancer | Binary | Questionnaire/Interview at recruitment | 20107 |
| Family history of cancer | Binary | Questionnaire/Interview at recruitment | 20107 |
| Family history of diabetes | Binary | Questionnaire/Interview at recruitment | 20107 |
| Smoking status | Binary | Questionnaire/Interview at recruitment | 20116 |
| Alcohol drinking status | Binary | Questionnaire/Interview at recruitment | 20117 |

| **Table S2. Participants' characteristics and effect of pancreatic cancer risk in the univariable logistic regression analysis.** | | | | | | |
| --- | --- | --- | --- | --- | --- | --- |
| Predictor | Entire study cohort^a^ | PCAND cohort^a^ | T2DM cohort^a^ | Missingness N (%) | Crude OR (95% CI) | P value |
| Overall | 12735 | 100 | 12635 | - | - | - |
| Anthropometry | | | | | | |
| Age at recruitment | 60(53-65) | 63(59-66) | 60(53-65) | 0 | 1.084(1.049-1.119) | 0.000 |
| Sex (Females) | 5131(40.3) | 37(37.0) | 5094(40.3) | 0 | 0.818(0.544-1.231) | 0.336 |
| Physical measure | | | | | | |
| Pulse rate | 73.0(64.5-81.5) | 71.5(65.0-78.0) | 73.0(64.5-81.5) | 740(5.8) | 0.991(0.976-1.008) | 0.300 |
| Diastolic blood pressure | 84.0(77.5-91.0) | 83.0(78.0-90.0) | 84.0(77.5-91.0) | 740(5.8) | 0.996(0.977-1.016) | 0.702 |
| Systolic blood pressure | 141.5(130.5-154.0) | 146.0(135.0-156.5) | 141.5(130.5-153.5) | 740(5.8) | 1.013(1.002-1.023) | 0.016 |
| Waist circumference | 102.0(94.0-112.0) | 103.0(95.0-110.0) | 102.0(94.0-112.0) | 45(0.4) | 1.002(0.988-1.017) | 0.783 |
| Weight | 89.0(77.6-101.7) | 88.9(79.4-101.4) | 89.0(77.6-101.7) | 83(0.7) | 1.001(0.991-1.012) | 0.806 |
| Body mass index (BMI) | 31.0(27.8-35.0) | 30.4(27.6-33.4) | 31.0(27.8-35.0) | 114(0.9) | 0.982(0.947-1.019) | 0.333 |
| Hip circumference | 107.0(102.0-115.0) | 107.0(102.0-112.0) | 107.0(102.0-115.0) | 49(0.4) | 0.994(0.976-1.012) | 0.516 |
| Standing height | 169.0(162.0-176.0) | 171.0(163.0-177.0) | 169.0(161.5-176.0) | 82(0.6) | 1.020(0.999-1.042) | 0.062 |
| Seated height | 139.0(133.0-143.0) | 140.0(135.0-144.0) | 139.0(133.0-143.0) | 89(0.7) | 1.016(0.987-1.046) | 0.276 |
| Sitting height | 89.0(86.0-93.0) | 91.0(87.0-94.0) | 89.0(86.0-93.0) | 89(0.7) | 1.038(0.998-1.079) | 0.061 |
| Blood biochemistry | | | | | | |
| Alanine aminotransferase | 26.8(19.5-37.5) | 26.5(20.7-36.5) | 26.8(19.5-37.5) | 626(4.9) | 0.999(0.988-1.010) | 0.837 |
| Albumin | 44.9(43.1-46.7) | 44.9(43.4-47.3) | 44.9(43.1-46.7) | 1550(12.2) | 1.026(0.950-1.108) | 0.513 |
| Alkaline phosphatase | 86.8(72.6-103.9) | 84.6(71.4-95.9) | 86.8(72.6-103.9) | 613(4.8) | 0.990(0.982-0.999) | 0.032 |
| Apolipoprotein A | 1.4(1.2-1.5) | 1.4(1.3-1.5) | 1.4(1.2-1.5) | 1592(12.5) | 1.025(0.440-2.388) | 0.954 |
| Apolipoprotein B | 1.0(0.8-1.2) | 0.9(0.8-1.1) | 1.0(0.8-1.2) | 742(5.8) | 0.729(0.335-1.588) | 0.426 |
| Aspartate aminotransferase | 26.1(21.9-32.6) | 26.2(23.3-31.4) | 26.1(21.9-32.6) | 670(5.3) | 1.002(0.993-1.012) | 0.624 |
| C-reactive protein | 2.4(1.2-4.8) | 2.1(1.1-3.8) | 2.4(1.2-4.8) | 647(5.1) | 0.949(0.897-1.004) | 0.070 |
| Calcium | 2.4(2.3-2.4) | 2.4(2.3-2.5) | 2.4(2.3-2.4) | 1553(12.2) | 3.881(0.495-30.445) | 0.197 |
| Cholesterol | 5.1(4.2-6.0) | 5.2(4.2-6.1) | 5.0(4.2-6.0) | 606(4.8) | 0.947(0.805-1.114) | 0.508 |
| Creatinine | 72.2(62.1-83.1) | 70.3(57.5-82.9) | 72.2(62.1-83.1) | 610(4.8) | 0.994(0.982-1.006) | 0.325 |
| Cystatin C | 1.0(0.9-1.1) | 0.9(0.8-1.0) | 1.0(0.9-1.1) | 608(4.8) | 0.862(0.309-2.406) | 0.777 |
| Direct bilirubin | 1.7(1.3-2.2) | 1.7(1.3-2.2) | 1.7(1.3-2.2) | 2409(18.9) | 1.130(0.965-1.323) | 0.129 |
| Gamma glutamyltransferase | 39.5(26.9-62.5) | 38.0(28.7-57.0) | 39.5(26.9-62.5) | 616(4.8) | 1.000(0.997-1.004) | 0.895 |
| Glucose | 5.7(5.0-7.1) | 5.5(5.0-6.7) | 5.7(5.0-7.1) | 1564(12.3) | 0.964(0.882-1.054) | 0.422 |
| Glycated haemoglobin (HbA1c) | 44.0(39.5-51.0) | 41.4(37.9-47.6) | 44.0(39.6-51.0) | 635(5.0) | 0.986(0.965-1.007) | 0.179 |
| HDL cholesterol | 1.2(1.0-1.4) | 1.1(1.0-1.4) | 1.2(1.0-1.4) | 1564(12.3) | 0.846(0.428-1.671) | 0.630 |
| IGF-1 | 19.5(15.5-23.6) | 19.5(16.0-23.3) | 19.5(15.5-23.6) | 671(5.3) | 0.990(0.958-1.024) | 0.574 |
| LDL direct | 3.1(2.5-3.8) | 3.2(2.4-3.9) | 3.1(2.5-3.8) | 641(5.0) | 0.894(0.719-1.110) | 0.309 |
| Lipoprotein A | 20.0(8.9-63.8) | 28.5(7.9-92.9) | 20.0(8.9-63.7) | 3316(26.0) | 1.004(1.000-1.008) | 0.060 |
| Oestradiol | 233.0(198.1-352.6) | 231.1(212.7-252.5) | 233.0(198.1-353.5) | 11285(88.6) | 0.993(0.981-1.005) | 0.253 |
| Phosphate | 1.1(1.0-1.3) | 1.1(1.0-1.2) | 1.1(1.0-1.3) | 1570(12.3) | 0.615(0.185-2.044) | 0.428 |
| Rheumatoid factor | 17.1(12.5-27.4) | 20.5(14.3-30.1) | 17.0(12.5-27.4) | 11687(91.8) | 1.008(0.977-1.039) | 0.629 |
| SHBG | 32.7(23.9-44.4) | 34.6(24.7-45.0) | 32.6(23.8-44.4) | 1642(12.9) | 0.997(0.986-1.007) | 0.541 |
| Testosterone | 7.8(1.3-11.0) | 8.3(1.4-10.9) | 7.8(1.3-11.0) | 1649(12.9) | 1.008(0.971-1.048) | 0.664 |
| Total bilirubin | 7.9(6.2-10.2) | 8.4(6.9-10.1) | 7.9(6.2-10.2) | 667(5.2) | 1.033(1.001-1.065) | 0.041 |
| Total protein | 72.7(70.1-75.6) | 73.2(71.0-76.0) | 72.7(70.0-75.6) | 1568(12.3) | 1.045(0.997-1.095) | 0.064 |
| Triglycerides | 2.0(1.4-2.9) | 2.0(1.5-3.0) | 2.0(1.4-2.9) | 626(4.9) | 1.044(0.901-1.210) | 0.567 |
| Urate | 341.2(288.6-399.1) | 356.6(304.8-402.9) | 341.0(288.6-399.0) | 619(4.9) | 1.001(0.999-1.003) | 0.437 |
| Urea | 5.4(4.6-6.4) | 5.5(4.6-6.1) | 5.4(4.6-6.4) | 615(4.8) | 0.959(0.841-1.094) | 0.533 |
| Vitamin D | 38.4(25.9-53.7) | 42.8(30.9-54.0) | 38.4(25.8-53.7) | 1165(9.1) | 1.008(0.998-1.018) | 0.102 |
| Blood count | | | | | | |
| SII | 531.7(387.2-728.3) | 563.7(406.3-708.7) | 531.7(387.1-728.5) | 440(3.5) | 1.000(0.999-1.000) | 0.632 |
| NLR | 2.2(1.7-2.8) | 2.4(1.8-2.9) | 2.2(1.7-2.8) | 440(3.5) | 1.106(0.988-1.237) | 0.081 |
| PLR | 118.5(93.1-150.0) | 112.9(87.3-139.9) | 118.6(93.2-150.1) | 440(3.5) | 0.997(0.993-1.001) | 0.189 |
| LMR | 4.2(3.2-5.4) | 4.2(3.2-5.0) | 4.2(3.2-5.4) | 443(3.5) | 0.999(0.954-1.046) | 0.968 |
| Basophill count | 0.03(0.0-0.05) | 0.03(0.01-0.05) | 0.02(0-0.05) | 440(3.5) | 2.465(0.165-36.877) | 0.514 |
| Basophill percentage | 0.41(0.3-0.65) | 0.40(0.29-0.60) | 0.41(0.30-0.65) | 440(3.5) | 0.868(0.535-1.411) | 0.569 |
| Eosinophill count | 0.19(0.1-0.26) | 0.19(0.10-0.27) | 0.19(0.10-0.26) | 440(3.5) | 1.559(0.523-4.650) | 0.426 |
| Eosinophill percentage | 2.3(1.5-3.4) | 2.3(1.5-3.8) | 2.3(1.5-3.4) | 440(3.5) | 1.040(0.944-1.146) | 0.428 |
| Haematocrit percentage | 41.9(39.4-44.4) | 42.3(40.2-44.0) | 41.9(39.4-44.4) | 415(3.3) | 1.037(0.982-1.096) | 0.194 |
| Haemoglobin concentration | 14.5(13.5-15.4) | 14.7(13.9-15.4) | 14.5(13.5-15.4) | 415(3.3) | 1.159(0.991-1.356) | 0.064 |
| High light scatter reticulocyte count | 0.023(0.017-0.031) | 0.022(0.017-0.031) | 0.023(0.017-0.031) | 615(4.8) | 10.814(0.000-100932956.3) | 0.771 |
| High light scatter reticulocyte percentage | 0.5(0.36-0.67) | 0.48(0.35-0.63) | 0.50(0.36-0.67) | 615(4.8) | 1.016(0.767-1.347) | 0.911 |
| Immature reticulocyte fraction | 0.32(0.28-0.36) | 0.31(0.27-0.35) | 0.32(0.28-0.36) | 615(4.8) | 0.030(0.001-0.776) | 0.035 |
| Lymphocyte count | 2.1(1.7-2.6) | 2.0(1.7-2.6) | 2.1(1.7-2.6) | 440(3.5) | 0.891(0.659-1.203) | 0.450 |
| Lymphocyte percentage | 28.3(23.5-33.3) | 26.5(22.9-32.3) | 28.3(23.5-33.3) | 440(3.5) | 0.975(0.949-1.003) | 0.076 |
| Mean corpuscular haemoglobin | 31.3(30.1-32.4) | 31.6(30.2-32.8) | 31.3(30.1-32.4) | 415(3.3) | 1.114(1.025-1.211) | 0.011 |
| Mean corpuscular haemoglobin concentration | 34.5(33.8-35.1) | 34.7(34.2-35.2) | 34.4(33.8-35.1) | 416(3.3) | 1.140(1.005-1.293) | 0.042 |
| Mean corpuscular volume | 90.5(87.6-93.4) | 91.2(88.1-94.0) | 90.5(87.6-93.4) | 415(3.3) | 1.039(0.999-1.080) | 0.053 |
| Mean platelet (thrombocyte) volume | 9.3(8.6-10.1) | 9.5(8.8-10.2) | 9.3(8.6-10.1) | 415(3.3) | 1.108(0.931-1.318) | 0.248 |
| Mean reticulocyte volume | 105.5(101.0-110.3) | 104.3(99.8-109.8) | 105.5(101.0-110.3) | 615(4.8) | 0.988(0.964-1.013) | 0.362 |
| Mean sphered cell volume | 81.6(78.4-85.0) | 81.3(78.5-85.3) | 81.6(78.4-85.0) | 615(4.8) | 1.015(0.978-1.053) | 0.423 |
| Monocyte count | 0.5(0.4-0.6) | 0.5(0.4-0.6) | 0.50(0.40-0.61) | 440(3.5) | 0.899(0.352-2.299) | 0.824 |
| Monocyte percentage | 6.8(5.5-8.2) | 6.8(5.5-8.2) | 6.8(5.5-8.2) | 440(3.5) | 0.972(0.890-1.061) | 0.526 |
| Neutrophill count | 4.5(3.7-5.6) | 4.7(3.9-5.7) | 4.5(3.7-5.6) | 440(3.5) | 1.085(0.962-1.223) | 0.185 |
| Neutrophill percentage | 61.4(55.8-66.7) | 63.8(57.5-67.5) | 61.4(55.8-66.7) | 440(3.5) | 1.020(0.996-1.045) | 0.103 |
| Nucleated red blood cell count | 0(0-0) | 0(0-0) | 0(0-0) | 441(3.5) | - | 0.999 |
| Nucleated red blood cell percentage | 0(0-0) | 0(0-0) | 0(0-0) | 441(3.5) | - | 0.999 |
| Platelet count | 246.0(208.0-287.7) | 225.7(188.4-265.3) | 246.1(208.0-287.9) | 415(3.3) | 0.994(0.991-0.998) | 0.001 |
| Platelet crit | 0.23(0.20-0.26) | 0.22(0.18-0.26) | 0.23(0.20-0.27) | 415(3.3) | 0.002(0.000-0.131) | 0.003 |
| Platelet distribution width | 16.5(16.2-16.9) | 16.6(16.3-17.0) | 16.5(16.2-16.9) | 415(3.3) | 1.412(0.999-1.996) | 0.051 |
| Red blood cell (erythrocyte) count | 4.6(4.4-4.9) | 4.7(4.4-4.9) | 4.6(4.4-4.9) | 415(3.3) | 1.038(0.650-1.658) | 0.875 |
| Red blood cell (erythrocyte) distribution width | 13.5(13.0-14.0) | 13.4(13.0-13.9) | 13.4(13.0-14.0) | 415(3.3) | 0.945(0.770-1.160) | 0.587 |
| Reticulocyte count | 0.072(0.056-0.092) | 0.070(0.056-0.088) | 0.072(0.056-0.092) | 615(4.8) | 5.262(1.308-21.160) | 0.019 |
| Reticulocyte percentage | 1.6(1.2-2.0) | 1.5(1.2-1.8) | 1.6(1.2-2.0) | 615(4.8) | 1.053(1.004-1.105) | 0.033 |
| White blood cell (leukocyte) count | 7.4(6.3-8.8) | 7.5(6.4-8.9) | 7.4(6.3-8.8) | 415(3.3) | 1.040(0.942-1.148) | 0.433 |
| Family history | | | | | | |
| Family history of bowel cancer | 1389(10.9) | 16(16.0) | 1373(10.9) | 0 | 1.571(0.916-2.692) | 0.101 |
| Family history of cancer | 4243(33.3) | 37(37.0) | 4206(33.3) | 0 | 1.186(0.788-1.784) | 0.414 |
| Family history of diabetes | 5035(39.5) | 38(38.0) | 4997(39.5) | 0 | 1.000(0.666-1.502) | 1.000 |
| Behavioral/lifestyle characteristics | | | | | | |
| Smoking status (never) | 5853(46.0) | 38(38.0) | 5815(46.0) | 108(0.8) | 0.698(0.465-1.048) | 0.083 |
| Alcohol drinking status (never) | 1091(8.6) | 3(3.0) | 1088(8.6) | 56(0.4) | 0.346(0.109-1.094) | 0.071 |

Note: ^a^ N (%) for dichotomous and median (IQR) for continous variables

| **Table S3. Summary results of SNPs used.** | | | | |
| --- | --- | --- | --- | --- |
| Order | SNP rsID | CHR | Position (GRCh37) | Minor Allele |
| 1 | rs79413112 | 1 | 89438600 | T |
| 2 | rs79183707 | 1 | 97123895 | A |
| 3 | rs75324536 | 2 | 11110270 | A |
| 4 | rs115959858 | 3 | 4597614 | A |
| 5 | rs12186013 | 3 | 73057450 | G |
| 6 | rs11922138 | 3 | 132521565 | G |
| 7 | rs75885323 | 3 | 132558168 | G |
| 8 | rs73205600 | 3 | 132642505 | G |
| 9 | rs78782961 | 4 | 157842922 | C |
| 10 | rs17796049 | 5 | 6839165 | G |
| 11 | rs4836103 | 5 | 123994762 | G |
| 12 | rs76673001 | 6 | 39897360 | A |
| 13 | rs17657500 | 6 | 122371683 | G |
| 14 | rs6465133 | 7 | 87850979 | C |
| 15 | rs45503495 | 7 | 99434215 | G |
| 16 | rs117271294 | 8 | 14071578 | A |
| 17 | rs75906400 | 10 | 58885379 | T |
| 18 | rs116860688 | 10 | 106157002 | T |
| 19 | rs75879855 | 10 | 107748133 | T |
| 20 | rs61759623 | 12 | 25401876 | C |
| 21 | rs116604830 | 12 | 111345219 | T |
| 22 | rs80211336 | 15 | 68886464 | A |
| 23 | rs72784921 | 16 | 22015346 | T |
| 24 | rs12151267 | 19 | 39453232 | G |

| **Table S4. The distribution of three risk groups among the entire population.** | | | |
| --- | --- | --- | --- |
|  | Low risk | Intermediate risk | High risk |
| Total | 11089 | 1392 | 254 |
| T2DM | 11063 | 1364 | 208 |
| PCAND | 26 | 28 | 46 |
| Event rate (%) | 0.23 | 2.01 | 18.11 |

| **Table S5. Genetic model diagnostic performance at different predicted probability cut-offs.** | | | | | | | |
| --- | --- | --- | --- | --- | --- | --- | --- |
| Probability cut-off | Distinct cut-offs* | |  | Common cut-offs | | | |
|  | 0.95% | 5.13% |  | 0.50% | 1% | 5% | 10% |
| Sensitivity | 0.790 | 0.470 |  | 0.860 | 0.760 | 0.470 | 0.280 |
| Specificity | 0.821 | 0.984 |  | 0.675 | 0.830 | 0.983 | 0.995 |
| PPV^a^ | 0.034 | 0.185 |  | 0.021 | 0.034 | 0.180 | 0.329 |
| NPV^b^ | 0.998 | 0.996 |  | 0.998 | 0.998 | 0.996 | 0.994 |
| Accuracy | 0.821 | 0.980 |  | 0.676 | 0.829 | 0.979 | 0.990 |
| Need screening | 0.183 | 0.020 |  | 0.329 | 0.178 | 0.020 | 0.007 |

^a^Positive predictive value, ^b^Negative predictive value

* The determination methods for the two distinct cut-offs are the same as those for the final model.

| **Table S6. The distribution of three risk groups among the entire population based on genetic model.** | | | |
| --- | --- | --- | --- |
|  | Low risk | Intermediate risk | High risk |
| Total | 10395 | 2086 | 254 |
| T2DM | 10374 | 2054 | 207 |
| PCAND | 21 | 32 | 47 |
| Event rate (%) | 0.20 | 1.53 | 18.50 |
